# Decarbonizing ammonia synthesis plants through retrofitting novel reformer technology

**DOI:** 10.1038/s41598-025-28598-y

**Published:** 2025-12-15

**Authors:** Tagwa Musa, Nada Mahmoud, Mohamed S. Challiwala, Eiman Mohamed, Hanif Choudhury, Nimir O. Elbashir

**Affiliations:** 1 Chemical Engineering Program, Texas A&M University at Qatar, Doha, 23874 Qatar; 2 College of Science and Engineering, Hamad bin Khalifa University, Doha, Qatar

**Keywords:** Ammonia production, Retrofitting, Dual reforming, CNTs, Decarbonization, Techno-economic analysis, Energy science and technology, Engineering, Environmental sciences

## Abstract

Decarbonizing ammonia production is crucial for reducing industrial greenhouse gas emissions; however, steam methane reforming (SMR) remains the dominant, carbon-intensive pathway. This study proposes a retrofit strategy for large-scale ammonia plants (1,268 tons/day) by replacing the conventional reformer with an advanced dual-reactor system that enables CO₂ utilization and carbon valorization. The novel configuration co-produces synthesis gas and multi-walled carbon nanotubes (MWCNTs), integrating ammonia and CNT production in a single process. Aspen Plus simulations compare the baseline SMR process with the retrofitted configuration, assessing energy demand, feedstock consumption, CO_2_ emissions, and economic performance. The retrofitted system achieves a 76% reduction in plant-level (Scops 1–1) CO₂-equivalent emissions and an 18.2% decrease in total specific energy (with a 31.5% reduction in thermal/steam duties), despite a 2.6-fold increase in methane input. At 25% MWCNT recovery, the Levelized Cost of Ammonia (LCOA) increases to $736.3/ton (vs. $308.3/ton for SMR); however, substantial co-product revenue yields a 3.7-fold increase in Net Present Value (NPV), 52% Internal Rate of Return (IRR), and a 4.5-year payback period. Sensitivity analyses support the robustness of the economic potential, confirming the viability of integrated CNT-ammonia production as a pathway for sustainable, low-carbon manufacturing.

**Supplementary Information:**

The online version contains supplementary material available at 10.1038/s41598-025-28598-y.

## Introduction

Ammonia (NH₃) plays a vital role in modern industry, primarily as a feedstock for nitrogen-based fertilizers and increasingly as a vector for hydrogen storage and energy transport due to its high hydrogen content, ease of liquefaction, and well-established infrastructure^1,2^. With over 180 million tons produced globally every year, ammonia production consumes substantial energy resources. It contributes significantly to global emissions, accounting for approximately 1.6% of total CO₂ emissions and 5% of emissions from the chemical industry^3–5^. This carbon intensity primarily stems from the hydrogen production step, typically achieved through steam methane reforming (SMR), which is both energy-intensive and reliant on fossil fuels^6–8^.

The traditional Haber–Bosch (HB) process, which reacts hydrogen and nitrogen under high pressures (150–350 bar) and temperatures (250–450 °C), remains the cornerstone of ammonia synthesis^7,9^. However, its extreme operating conditions and low per-pass conversion rates (~ 24% nitrogen conversion) impose substantial energy and capital demands^10–12^. The hydrogen feedstock, typically derived from natural gas or coal, is responsible for the majority of CO₂ emissions, with SMR-based production resulting in 2.5–2.9 kg CO₂-eq per kg of NH₃. At the same time, coal-based gasification, common in China, produces up to 5.2 kg CO₂-eq/kg NH₃^13^.

Decarbonization of ammonia production is a critical goal in the transition to sustainable chemical processes. Multiple approaches have been explored. One route focuses on improving catalyst efficiency under milder synthesis conditions. Recent work on inverse-structured iron-based catalysts has achieved triple the volumetric activity of conventional Fe systems, enabling ammonia synthesis at temperatures as low as 50°C^14^. Ruthenium-based catalysts on supports such as La₂O₃ and CeO₂, often promoted with alkali metals, have demonstrated high activity at moderate pressures and temperatures^15,16^. Although promising, these catalysts remain largely at the experimental stage. Additionally, nanostructured supports and dual-oxide systems, such as MgO–Nd₂O₃, exhibit enhanced selectivity and thermal stability; however, their commercial application remains limited^17^.

Process-level innovations have targeted reduced operating pressure and energy consumption through selective ammonia absorption using metal salts and tailored adsorption systems, enabling recycling at pressures as low as 20 bar^9,16^. These systems offer alternatives to conventional condensation-based separation, which demands higher pressure and refrigeration energy.

Ammonia is also classified based on the source of hydrogen used in its production, leading to descriptors such as grey, blue, and green ammonia. Due to cost advantages, grey ammonia, produced from fossil-derived hydrogen without carbon capture, remains the most prevalent. In contrast, blue ammonia incorporates carbon capture and storage (CCS), reducing net CO₂ emissions to about 0.856 kg CO₂-eq/kg NH₃ but increasing capital and operating costs^18,19^. This increase is partially attributed to the energy penalty of CCS systems, which typically ranges between 15% and 20%, depending on capture technology and integration level^18^. Green ammonia, which relies on hydrogen produced from renewable-powered electrolysis, offers the lowest emissions (~ 0.052 kg CO₂-eq/kg NH₃) but is currently constrained by high energy costs, limited electrolyzer efficiency, and challenges in hydrogen compression^9,20,21^. Even with green hydrogen retrofits, maximum CO₂ reductions in existing grey plants are limited to ~ 10% due to integration and thermal constraints^20^.

Alternative technologies have also been evaluated within the reforming step, which is the most emissions-intensive component of ammonia production. Partial oxidation (POX) and autothermal reforming (ATR) offer exothermic or thermally balanced alternatives to SMR^14,22,23^. Dry reforming of methane (DRM), which utilizes CO₂ as an oxidant, is attractive in theory but suffers from high coke formation, unfavorable syngas ratios, and excessive energy demand^23,24^. Hybrid approaches, including tri-reforming and membrane-assisted air separation, have demonstrated improved syngas quality and emission reductions^25,26^. For example, a 27% increase in ammonia output and a decrease in CO₂ emissions to near-zero levels were achieved through such integration^25^.

A more recent innovation in reforming technology involves integrating CO₂ utilization and carbon valorization within a single process unit. One such configuration is a dual-reactor system in which CH₄, CO₂, and O₂ are partially oxidized at moderate temperatures (~ 550–620 °C) to generate syngas while simultaneously precipitating solid carbon in the form of multi-walled carbon nanotubes (MWCNTs). The product gas is subsequently processed in a high-temperature reformer to adjust the H₂/CO ratio for synthesis applications. This approach eliminates the need for steam, reduces oxygen demand, and enables autothermal operation, thereby improving thermal integration. Experimental and simulation studies have demonstrated that such systems can reduce CO₂ emissions by over 70% in certain gas-to-liquid (GTL) applications and enhance overall energy efficiency and process economics compared to conventional reforming routes like DRM and ATR^27,28^.

Building on these advances, the present study evaluates the integration of such a carbon-valorizing reforming system into a conventional ammonia plant. A specific case study is presented, utilizing a dual-reactor reforming configuration, to assess energy use, lifecycle CO₂ emissions, and techno-economic performance in comparison to a conventional SMR-based facility. Steady-state simulations are conducted using Aspen Plus for a 1,268 ton/day ammonia plant, and the analysis includes co-production of CNTs alongside ammonia. Economic metrics, such as the Levelized Cost of Ammonia (LCOA), Net Present Value (NPV), Internal Rate of Return (IRR), and payback period, are evaluated under conservative assumptions, with sensitivity analyses conducted across ammonia, carbon credit, CNT, and natural gas prices. The study aims to explore the feasibility of integrated reforming-based retrofits as a potential decarbonization pathway that combines emissions reduction with process intensification and value co-creation.

## Methodology

This study presents a comparative simulation-based assessment of ammonia production using two reforming technologies: the conventional Steam Methane Reforming (SMR) process and a dual-reactor reforming system capable of CO₂ utilization and carbon valorization. A specific dual-reforming configuration, hereafter referred to as the “retrofitted configuration”, is examined as a case study to evaluate the feasibility and performance of an integrated ammonia and carbon product pathway.

### Simulation framework and process modeling

**Fig. 1:**
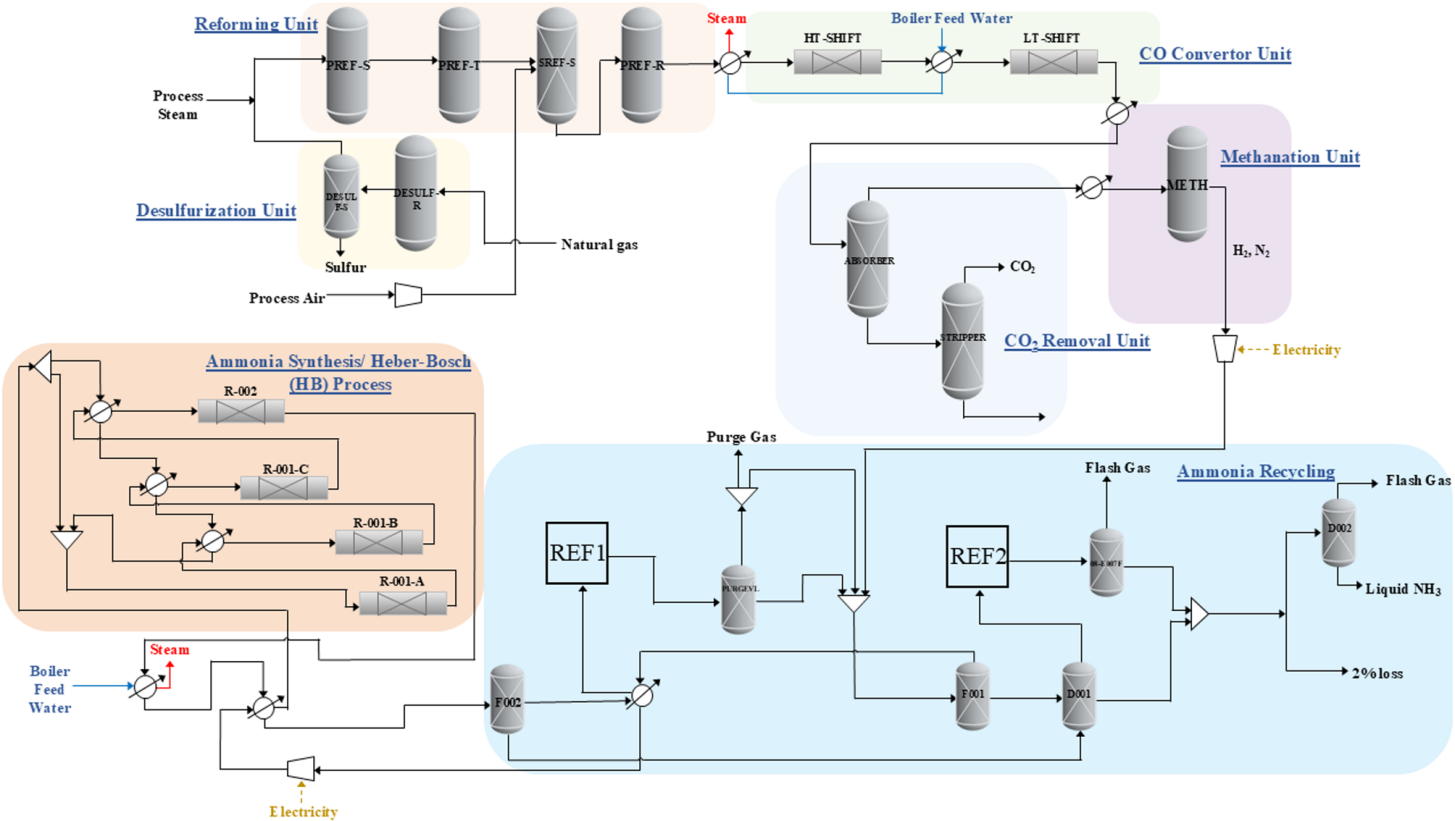
SMR-based ammonia simplified process flow diagrams.

All process simulations were developed using Aspen Plus V12.1 under steady-state conditions, replicating the main stages of an industrial ammonia plant with a nominal production capacity of 1270 metric tons per day. The conventional SMR-based configuration includes feed desulfurization, primary and secondary reforming, high- and low-temperature water-gas shift (WGS), CO₂ removal, methanation, and ammonia synthesis via the Haber–Bosch loop. A simple base-case flowsheet is illustrated in Fig. 1, while the completed detailed flow sheet is shown in Figure S1a in the supplementary Information (SI). This model was validated against benchmark data^29^, ensuring consistency in mass and energy balances. The plant capacity (1,268 ton NH₃/day) was adopted from Lee et al.^29^, who report a conventional natural gas-based ammonia plant at 1,270 ton/day, serving as the validation baseline for the present model.

Both the SMR and the retrofitted configuration were simulated using the Redlich-Kwong-Soave equation of state with the Boston-Mathias alpha function (RKS-BM), which provides reliable predictions on thermodynamic properties at elevated pressures and temperatures^30,31^. To enhance accuracy in the ammonia synthesis loop, additional modifications were applied to improve vapor–liquid equilibrium and enthalpy predictions.

For the retrofitted configuration case, equilibrium-based Gibbs reactors (RGibbs) were employed to simulate the dual-reactor system. This approach is supported by experimental results^32^, which demonstrated near-equilibrium conversions with deviations of less than ± 8% for CH₄ conversion and consistent MWCNT yields across multiple scales. The reactor design utilizes a co-fed mixture of CH₄, CO₂, and O₂ at moderate temperatures (500–650 °C) and high pressure to promote rapid syngas formation and carbon nucleation under thermodynamically favorable conditions^28,32^. The modeling framework follows the CARGEN two-stage reforming concept previously validated for gas-to-liquid applications^27,28^ and adapted here for ammonia synthesis; the specific operating envelopes, reaction set, and integration logic are detailed in Sect. "Retrofitted configuration with dual-reactor reforming".

### Mass and energy balance reporting

For both configurations, detailed mass- and energy-balance data were extracted directly from the Aspen Plus V12.1 simulations. Stream tables include flow rates, compositions, temperatures, and pressures for each major unit operation, normalized per kilogram of ammonia produced at the fixed plant capacity of 1,268 tons of NH₃ per day.

Energy balances were obtained from the model’s equipment and utility reports. Electrical power (compressors, pumps, auxiliaries) and thermal duties (reactors, heat exchangers, and heaters) were exported in kW and converted to kWh per kg NH₃ by dividing by the hourly ammonia production rate. Total specific energy consumption equals the sum of electricity and steam-equivalent heat. The steam contribution is reported as energy (kWh/kg) rather than as a separate mass flow, consistent with the plant’s utility model.

Comprehensive stream summaries for both the conventional SMR and retrofitted configurations are presented in Tables S1 and S2 (SI), and the unit operation energy breakdown is shown in Table S3.

### Base case SMR configuration

The SMR-based ammonia plant model represents a conventional facility that utilizes natural gas. The feed composition (Table 1) is primarily composed of methane (80.0%), along with 17.7% ethane, 1.25% heavier hydrocarbons (C₃+), and trace components, including nitrogen, oxygen, and sulfur compounds. The gas enters the system at 45 °C and 38.2 bar and is first processed in a desulfurization unit to protect downstream catalysts^29^.

**Table 1:** Natural gas feed composition and Inlet Conditions^29^.

Component	Mole fraction (%)
CH_4_	80.0
C_2_H_6_	17.7
C_3_+	1.25
N_2_	0.8
O_2_	0.2
Sulfur Compounds	0.0001
Temperature (^o^C)	45
Pressure (bar)	38.2

Following desulfurization, the feed is mixed with steam and introduced into a primary reformer operating at 791 °C and 30.7 bar, where partial conversion to H₂, CO, and residual CH₄ occurs over Ni-based catalysts. The resulting gas is then fed to a secondary reformer with preheated air, facilitating complete methane conversion and the addition of nitrogen in a stoichiometric proportion. Table 2 presents the composition of the secondary reformer outlet.

**Table 2:** Secondary reformer outlet stream Properties^29^.

Component	Mole fraction (%)
H_2_	35.5
H_2_O	35.3
N_2_	15.2
CO	8.4
CO_2_	5.1
CH_4_	0.3
Ar	0.2
Temperature (°C)	980
Pressure (bar)	29

The syngas undergoes WGS to reduce CO content from 8.4% to approximately 0.2%, followed by CO₂ removal using amine scrubbing to achieve < 0.3% CO₂. The purified stream, with a near-ideal 3:1 H₂:N₂ ratio, proceeds to methanation, where trace CO and CO₂ are converted to CH₄ and H₂O. The resulting feed to the ammonia synthesis loop consists of ~ 74% H₂, ~ 25% N₂, and < 1% CH₄.

Ammonia synthesis occurs at ~ 292 bar via the Haber–Bosch loop, with single-pass nitrogen conversion of ~ 24%. Reactor effluent contains unreacted H₂, N₂, and ~ 24% NH₃, reflecting equilibrium-limited performance.

### Retrofitted configuration with dual-reactor reforming

**Fig. 2:**
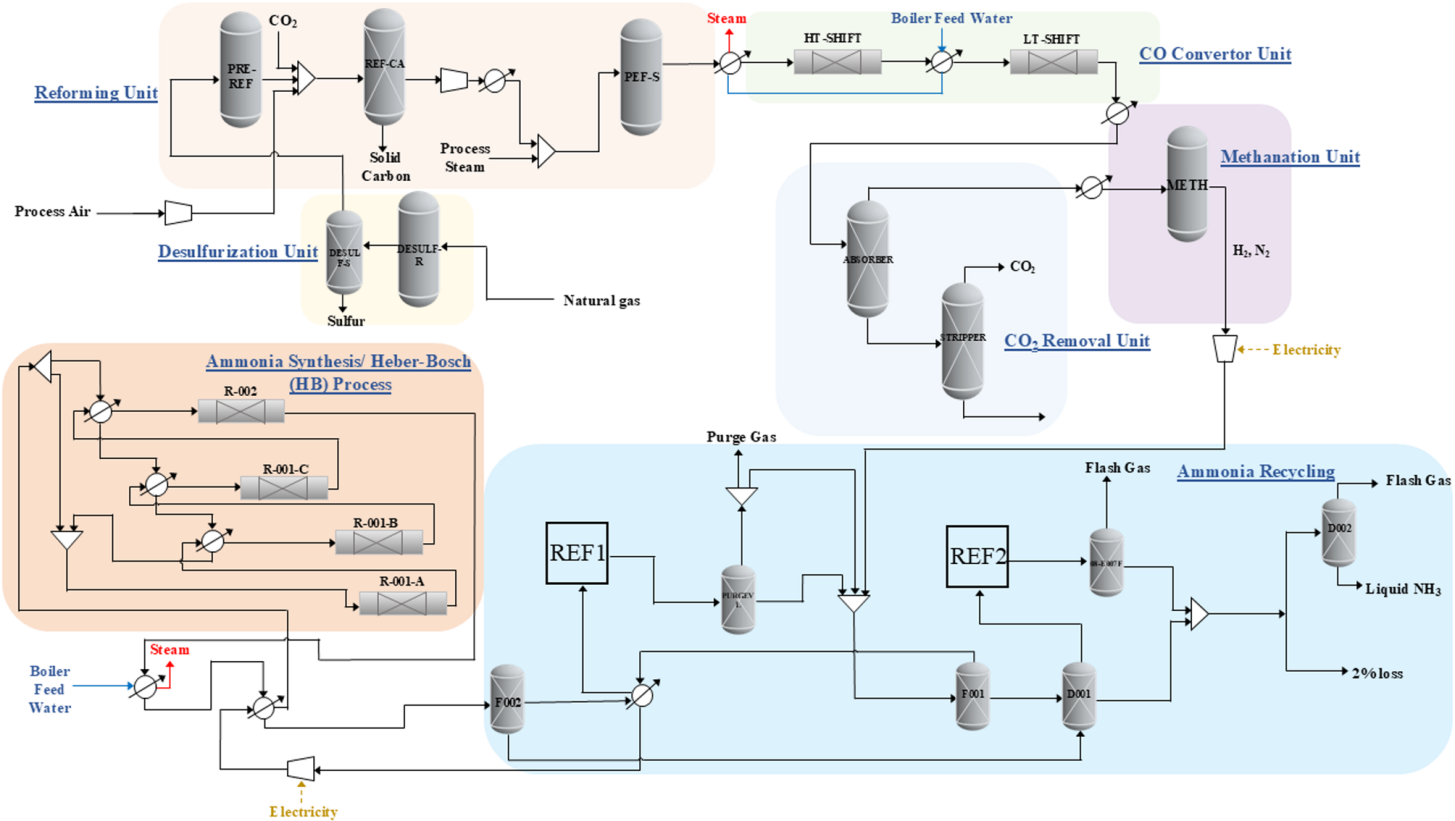
Simplified process flow diagrams for retrofit configuration.

In the retrofitted configuration, the conventional SMR and secondary reformers are replaced by a dual-reactor reforming system that simultaneously utilizes CO₂ and valorizes carbon (Fig. 2; Figure S1b). The first reactor operates at 400–650 °C and atmospheric pressure, where CH₄, CO₂, and O₂ (in a molar ratio of 1:0.6:0.1) are partially oxidized to produce syngas and a solid carbon intermediate. The second reactor, operating at elevated temperature, finalizes the syngas composition. The retrofitted system is tuned to match the H₂:N₂ ratio of the base-case outlet, ensuring seamless integration with downstream process units.

*Applicability of prior GTL studies to ammonia*: The dual-reactor configuration is adapted from CARGEN reactor concepts originally demonstrated for gas-to-liquid processes^27,28^, however, the process objectives and integration scheme are redefined for ammonia production. Reactor 1 is maintained in the carbon-forming window (400–600 °C, ≈ 25 bar) to generate MWCNTs and syngas, while Reactor 2 is operated at 750–820 °C and 20–30 bar to increase the H₂ fraction and reduce CO. The target ratio after cleanup is H₂-rich (greater than 3:1 over CO), after which CO/CO₂ are removed and N₂ is blended to achieve the H₂:N₂ ≈ 3:1 feed for the Haber–Bosch loop. These adjustments replace the Fischer–Tropsch targets of the original studies and ensure compatibility with ammonia synthesis.

The relevant equilibrium reactions considered in the Aspen simulation are:

CH₄ + CO₂ ⇌ 2 CO + 2 H₂; CH₄ + ½ O₂ → CO + 2 H₂; CH₄ → C(s) + 2 H₂; 2 CO ⇌ C(s) + CO₂; CH₄ + H₂O ⇌ CO + 3 H₂; CO + H₂O ⇌ CO₂ + H₂.

These reactions reproduce the reported experimental performance, where experimental conversions were within 10–15% of equilibrium, and carbon was confirmed as MWCNTs on Ni/Al₂O₃ catalysts^28^. The same reaction network is retained here for thermodynamic equilibrium modeling of both Reactor 1 and Reactor 2.

Simulation results estimate a carbon yield of 0.76 kg of MWCNTs per kg of NH₃ produced, corresponding to a theoretical annual output of 318,000 tons. To reflect commercialization constraints and avoid overestimation, a conservative 25% recovery factor is applied in the economic analysis, consistent with previous studies on similar reforming configurations^28,33^.

### Key modeling assumptions

To streamline comparative analysis, several simplifications were adopted. All process streams were modeled as single-phase with constant specific heat capacities. Phase transitions, latent heat effects, and pressure drops were neglected, a standard practice in early-stage feasibility studies for gas-phase systems^34,35^.

Reaction kinetics for SMR and WGS were taken from Aspen Plus libraries and validated against literature^29,30^. For the dual-reactor retrofitted configuration, the reformers were modeled using RGibbs modules based on published evidence indicating that equilibrium conversion accurately represents system performance under the studied operating conditions^27,28,32^.

### Performance evaluation metrics

#### Process performance

Four Key Performance Indicators (KPIs) were defined:

1. Natural Gas Consumption (kg/kg NH₃),
2. Steam Demand (kg/kg NH₃),
3. Total Energy Use, including heat duties and compression work (kWh/kg NH₃),
4. CO₂ Emissions, direct (Scope 1) and indirect (Scope 2).

#### CO₂ emissions estimation

Direct emissions originate from reforming reactions and combustion. Indirect emissions associated with utilities were estimated using a baseline emission factor of 0.36 kg CO₂/kWh, derived from Aspen Plus simulations of natural gas-fired utilities. All emissions are reported in kg CO₂-eq per kg NH₃ using GWP100 metrics. While this factor provides internal consistency, regional variations (0.05–0.8 kg CO₂/kWh) are acknowledged as a source of uncertainty, particularly relevant for the retrofitted configuration, which exhibits higher electricity demand due to the additional process units^19^. A gate-to-gate boundary is applied at the plant battery limits. “Direct” refers to net process/combustion CO₂ (Scope 1); “indirect” refers to electricity/steam utilities (Scope 2). Process-step attribution for Scope 2 uses Aspen equipment and duty reports (air/NH₃/CO₂/retrofitting scenario compressors; reactor and heat-exchanger duties). Negative direct values can occur when carbon is retained as a solid product at battery limits; no end-of-life credit is taken, and long-term permanence is not assumed. The 0.36 kg CO₂ per kWh factor is applied to both electric power and steam-equivalent thermal energy, consistent with on-site natural-gas-fired utilities. Steam duties are converted to kWh thermal and multiplied by this factor. For the HP steam grade used (Δh ≈ 2.9 GJ/ton), the implied steam emission factor is approximately 290 kg CO₂/ton of steam.

Oxygen supply is treated as follows: the central case assumes purchased O₂, and an on-site air separation unit (ASU) sensitivity is provided in the SI using a cryogenic specific power of 220 kWh per ton O₂ (200–400 kWh per ton O₂) to quantify the incremental Scope 2 burden. Upstream emissions (e.g., natural-gas production and fugitive CH₄) are excluded from the base inventory and reported as a lifecycle assessment (LCA) sensitivity in the SI using IPCC AR6 GWP₁₀₀ (fossil CH₄) and leakage ranges from recent empirical studies^36,37^.

### Economic assessment framework

#### Assumptions and input parameters

The economic evaluation is based on a 30-year project horizon and an 8% real discount rate. A nominal production of 1,268 tons NH₃/day (418,440 tons/year) was assumed. Input values are summarized in Table 3.

**Table 3:** Economic input parameters for Techno-Economic Analysis.

Parameter	Value	Reference
Natural gas	$4.0/MMBtu (baseline)	^38^
Oxygen (retrofitted configuration only)	$100/ton	^39^
CO₂ feed (retrofitted configuration only)	$50/ton	^40^
Electricity	$0.07/kWh	^41^
Steam (HP, on-site; derived*)	$12.2/ton steam	This work
Carbon-credit revenue	$30/ton CO₂ (baseline)	^42,43^
Ammonia selling price	$450/ton (baseline)	^44^
MWCNTs Selling Price	$5/kg (baseline)	^45^

*Steam is generated on site; the listed steam unit cost is a derived on-site value and is not a separate purchase (see SI for basis).

#### CAPEX and OPEX estimation

*CAPEX (SMR baseline)*: The SMR plant CAPEX was back-calculated from the literature LCOA reported in 2022 by Lee et al.^29^ at the validated scale, using Eq. (1) and the study’s financial assumptions (8% real, 30 y) and plant rate (1,268 tons NH₃/day). The numerical substitution is provided in Table S4 (SI); the resulting CAPEX is reported in 2022 USD. The implied capital charge and the corresponding OPEX per ton are consistent with the natural-gas-dominated cost structure reported in 2022 by Lee et al.^29^.

*CAPEX (retrofitted configuration)*: a + 50% uplift relative to the SMR total CAPEX (equal capacity and cost year) is adopted as the central estimate, reflecting added dual-reactor hardware and associated utilities (carbon-forming R1, high-T R2, CO₂ compression/recycle, MWCNT handling, interconnects) and supported by the two-reactor analogue^28^. Scope mapping (displaced vs. added inside battery limits (ISBL)/outside battery limits OSBL) and parameter values are documented in Table S5 (SI). The choice of oxygen supply is treated as a scenario (purchased O₂ vs. on-site ASU) with assumptions defined in Sect. "Uncertainty and sensitivity methodology" and Table S6a (SI).

*OPEX (definition and baselines)*: OPEX comprises feedstock, utilities, chemicals/consumables, operating labor/staff, and maintenance (i.e., manufacturing cost excluding capital charges and non-plant overheads). Maintenance is 3% of fixed-capital investment (FCI) per year (within the 2–10% FCI range). Operating labor/staff is 10% of total OPEX (consistent with operating labor of 10–20% of manufacturing cost)^46^. For the retrofitted configuration, a chemicals/consumables adder of 3% of total OPEX is included by analogy to the dual-reactor GTL implementation^28^. Carbon credit and MWCNT revenues are included in discounted cash-flow metrics (NPV/IRR).

Steam is generated on-site in a natural-gas-fired boiler, so it is not purchased as a separate utility. The steam unit cost listed in Table 3 is a derived on-site value (based on the natural-gas price and boiler efficiency) and is not double-counted with the natural-gas line.

#### Economic performance indicators

The economic viability of both ammonia production configurations was evaluated using LCOA, NPV, and IRR over a 30-year project lifetime, assuming no salvage value was available.

LCOA was computed using^47^:

1 $$\:LCOA=\frac{CRF\times\:CAPEX+Annual\:OPEX}{Annual\:N{H}_{3}Production\:\left(tons\right)}$$

where the Capital Recovery Factor (CRF) is defined as:

2 $$\:CRF=\frac{r{(1+r)}^{n}}{{(1+r)}^{n}-1}$$

where: r is the discount rate (8%), and n is the project lifetime (30 years).

The NPV was calculated using the discounted cash flow approach:

3 $$\:NPV=\sum\:_{t=1}^{n}\frac{{R}_{t}-{C}_{t}}{{(1+r)}^{t}}-CAPEX$$

where R_t_ and C_t_ are the total revenues and costs in year t, respectively. CAPEX is treated as an upfront investment at year t = 0.

For the SMR baseline, CAPEX is from the back-calculation described in Table S4. For both configurations, LCOA is computed via Eq. (1) using the OPEX and prices specified in Table 3.

Inflation rates were differentiated by revenue or cost type, by sector-specific economic trends:

- A 2% annual inflation was applied to ammonia prices, reflecting historical behavior in the nitrogen fertilizer sector^48^.
- A 2.5% escalation rate was applied to operating expenditures (OPEX), capturing increases in energy, chemicals, and labor costs^49^.
- A 1% inflation rate was used for carbon credit revenues, accounting for volatility in carbon pricing and regulatory uncertainty^50^.

The IRR, defined as the discount rate at which NPV equals zero^51^, was also computed to assess investment attractiveness. The IRR values were calculated using Microsoft Excel’s built-in financial functions based on the annual net cash flow profiles. Python was used exclusively for generating figures and visualizing economic trends.

#### Uncertainty and sensitivity methodology

*Market-price parameters*:

- Ammonia product price: $300–$800/ton NH₃.
- MWCNT selling price: $2–$80/kg.
- Carbon credit price: $30–$100/ton CO₂.
- Natural gas price: $1.5–$13/MMBtu (captures low-cost, average, and high-import conditions).

*Structural/design cost parameters for retrofit case*:

- Retrofit CAPEX uplift (vs. SMR total CAPEX): +35–65% (central + 50%).
- Maintenance: 1.5–4.5% of fixed-capital investment per year (central 3%).
- Operating labor/staff: 5–15% of total OPEX (central 10%).
- Chemicals/consumables: 0–6% of total OPEX (central 3%).
- Oxygen-supply scenario: Case A (purchased O₂, central case) versus Case B (on-site ASU). For the ASU case, a cryogenic specific power of 220 kWh/ton O₂ is used, and a CAPEX adder of 10% of the SMR total CAPEX is applied^52^. Assumptions and calculation details are provided in Table S6a.

## Results and discussion

A comparative analysis was conducted between the base-case SMR-based ammonia production process and the retrofitted configuration featuring a dual-reactor reforming system for integrated CO₂ utilization and carbon valorization. The comparison focuses on process efficiency, feedstock, energy consumption, and carbon footprint. All simulations were performed under steady-state conditions using Aspen Plus V12.1, with model validation against benchmark literature to ensure the reliability of the results.

### Simulation validation and reforming process comparison

The SMR-based ammonia plant simulation was validated for mass and energy balances against the reference study^29^. As detailed in Table 4, the differences are less than 3% for most unit operations, including the SRM outlet, the WGS section, and the CO₂ removal unit. Larger discrepancies (20–30%) were observed in internal synthesis loop flows, particularly in the recycle feed, reactor inlet gas, and purge stream.

These variances are attributed to modeling choices and system sensitivities specific to high-pressure ammonia synthesis loops. Slight differences in CO₂ removal efficiency or inert gas content (e.g., Ar, CH₄) downstream of purification significantly influence hydrogen-to-nitrogen ratios, recycle rates, and purge volumes. Furthermore, assumptions related to vapor–liquid separation, purge ratios, and simplified modeling of the final methanation step for CO/CO₂ removal may introduce compounding effects in loop circulation, without materially affecting ammonia yield or syngas quality.

These details are well-documented in simulation studies of ammonia synthesis systems, where internal loop behavior exhibits a nonlinear dependency on purge and separation efficiency^53^. Despite differences, the present model captures all critical performance metrics, natural gas consumption, steam demand, syngas composition, and ammonia production, within an acceptable range, confirming its robustness for comparative analysis.

**Table 4:** Selected validation metrics comparing simulation results with benchmark data^29^.

Stream	Benchmark data^29^ (kg/kg NH₃)	Present study (kg/kg NH₃)	% Deviation
Process NG	0.52	0.52	0%
Steam Input	1.93	1.93	0%
SRM Outlet	3.76	3.76	0%
WGS Outlet	3.76	3.76	0%
CO₂ Removal Outlet	1.16	1.19	–3%
Recycle Feed	2.47	2.97	–20%
Reactor Inlet Gas	3.37	4.18	–24%
NH₃ Product	-	1.00	—

The reforming section is the most energy- and carbon-intensive stage of ammonia production, serving as the focal point for retrofitting. In the SMR-based configuration, the primary and secondary reformers produce a syngas stream comprising 35.5% H₂, 15.2% N₂, and 5.1% CO₂ at 980 °C and 29 bar. In contrast, the dual reactor retrofitted configuration, which utilizes CH₄, CO₂, and O₂, was tuned to achieve a similar syngas composition, eliminating the need for steam injection.

The specific case study presented here achieved thermodynamic equilibrium for syngas generation while also enabling the co-production of MWCNTs, offering both environmental and potential economic benefits. The simulation results were validated against published experimental and scale-up studies^28,32^, confirming the reliability of the equilibrium-based approach. Key metrics, including CH₄ and CO₂ conversion, syngas composition, and carbon yield, matched closely with experimental data, as shown in Table 5.

**Table 5:** Thermodynamic validation of the retrofitted configuration simulation against experimental Data.

Parameter	Aspen plus simulation (This Study)	Experimental/model data^28,32^
CH₄:CO₂:O₂ Feed Ratio	1:0.6:0.1	1:0.6:0.1
Reactor 1 Temperature (°C)	550	500–600
CO₂ Conversion (%)	66	~ 65
CH₄ Conversion (%)	82	± 8% deviation from experimental
H₂/CO Ratio	1.9	~ 2.0
Carbon Product Yield (kg/kg NH₃)	0.76	Consistent multi-scale yield
Energy Reduction vs. DRM	50%	50%

### Energy and feedstock utilization

The integration of the dual-reactor reforming case study significantly altered the feedstock profile and energy requirements of the ammonia plant. As summarized in Table 6, the retrofit configuration consumed approximately 1.36 kg of natural gas per kg of ammonia, representing a 161.5% increase compared to the 0.52 kg/kg NH₃ consumption in the conventional SMR-based process. This increase is attributed to the additional methane feedstock required in the first reactor, which supports not only syngas formation but also the production of carbon nanotubes.

Despite the elevated methane input, the specific energy consumption of the retrofitted configuration decreased by 18.2% when electricity and thermal (steam-equivalent) contributions are reported: from 5.06 to 4.14 kWh/kg NH₃ (Table 6). This net reduction arises because the retrofit lowers thermal (steam/firing) demand, from 4.516 to 3.095 kWh/kg NH₃, by eliminating the fired reformer box and leveraging autothermal heat release and tighter heat integration, while compressor electricity increases from 0.544 to 1.043 kWh/kg NH₃ due to the addition of CO₂ and retrofit compression.

In addition, the partial conversion of methane into a solid MWCNT product reduces the carbon sent to combustion, and the 25% reduction in steam demand contributes substantially to the system’s improved thermal efficiency.

Nitrogen supply and synthesis conditions remain matched to Haber–Bosch requirements in both cases: air addition to the secondary reformer for the SMR baseline, and a dedicated N₂ stream (ASU) or equivalent utility blending for the retrofit, maintaining an H₂:N₂ ratio of 3:1 at a constant plant capacity of 1,268 tons per day.

**Table 6:** Key process inputs and specific energy use (per kg NH₃).

Parameter	SMR configuration	Retrofitted configuration	Change (%)
Natural Gas Consumption (kg/kg NH₃)	0.52	1.36	161.5
Steam Demand (kg/kg NH₃)	1.93	1.45	−24.9
Electricity (kWh/kg NH₃)	0.544	1.043	91.7
Thermal/steam-equivalent (kWh/kg NH₃)	4.516	3.095	−31.5
Total energy use (kWh/kg NH₃)	5.06	4.14	−18.2
Air/N_2_ supply (normalized)	Air to secondary reformer	Dedicated N₂ blending	-
NH₃ Yield (tons/day)	1,268	1,268	matched

Note: Thermal (steam-equivalent) aggregates positive process heat duties (reactors/heaters/heat-exchanger net inputs) and steam demand expressed on a kWh thermal basis. Steam conversion: 1 kg steam ≈ 0.806 kWh thermal (Δh ≈ 2.9 GJ per t HP steam); thus E_steam = 0.806 × (kg steam/kg NH₃). See Tables S1–S3 for unit-operation breakdowns.

### Carbon footprint reduction

**Fig. 3:**
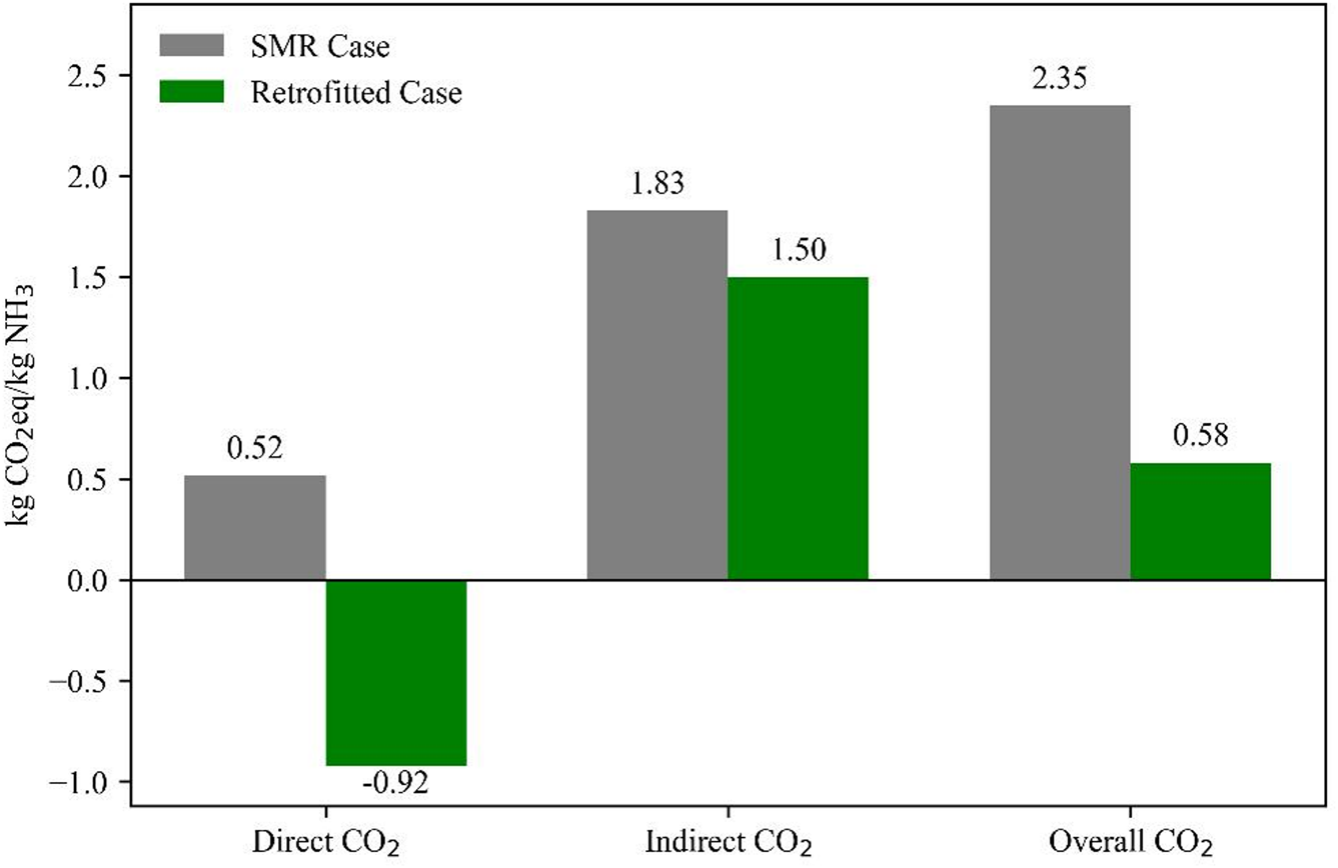
Breakdown of CO₂-equivalent emissions for SMR and the retrofitted configuration case modeled in this study.

The primary environmental advantage of retrofitting with an integrated reforming system lies in its substantial reduction of GHG emissions. As shown in Fig. 3, the total CO₂-equivalent emissions for the conventional SMR-based ammonia process were estimated at 2.35 kg CO₂-eq per kg NH₃, consistent with literature-reported values for large-scale natural gas-based ammonia plants without carbon capture^19^. In contrast, the retrofitted configuration modeled in this study achieved a 76% reduction, lowering net emissions to 0.58 kg CO₂-eq/kg NH₃. A process-step and scope breakdown is provided in Table S7 (SI), which reports compressor-level electricity, reactor and heat-exchanger thermal duties, and the net direct (battery-limits) CO₂ for each configuration (Case A: purchased O₂).

This emissions profile surpasses typical “blue ammonia” pathways, which combine SMR with carbon capture and storage (CCS) and report residual emissions in the range of 0.85 to 1.0 kg CO₂-eq/kg NH₃^18,19^. Such findings suggest that integrated reforming configurations with carbon valorization, as demonstrated in this case study, may serve as viable alternatives for achieving deep decarbonization in ammonia production, particularly when assessed within LCA frameworks and emerging carbon intensity benchmarks.

The observed reduction in carbon intensity is attributed to two synergistic mechanisms within the retrofit case:

- CO₂-reactive reforming chemistry: The first reactor operates under conditions that favor partial oxidation and dry reforming, where CO₂ acts as a chemical reactant, thereby reducing its release to the atmosphere.
- Solid Carbon formation: A portion of the carbon in the methane feed is transformed into MWCNTs. At the plant battery limits, this carbon remains in a solid, non-vented form and is counted as negative direct (Scope 1) CO₂. Long-term permanence depends on product fate and service life; accordingly, carbon retained in MWCNTs is not assumed to be permanently stored beyond the plant boundary.

Under the simulation assumptions, direct (Scope 1) emissions are − 0.92 vs. + 0.52 kg CO₂/kg NH₃ (retrofit vs. SMR). Indirect (Scope 2) emissions are 1.50 vs. 1.83 kg CO₂/kg NH₃, reflecting lower total utility demand in the retrofit (autothermal heat release and improved heat integration reduce thermal requirements; added compression does not offset this benefit).

Despite the higher natural-gas feed in the retrofit, indirect emissions are lower because total utility demand is smaller. Autothermal heat release in the dual-reactor system, along with improved heat recovery, reduces the overall thermal requirement. The added compressor loads do not offset this effect, resulting in a net decrease in electricity- and steam-related Scope-2 CO₂.

Upstream/fugitive methane is not included in the base inventory. To contextualize its potential impact, an LCA sensitivity (Table S8) applies GWP₁₀₀ (AR6, fossil CH₄ = 27.2) to leakage rates of 1–3%^36,37^. Using the plant CH₄ throughput as a proxy, this adds approximately 0.14 to 0.42 kg CO₂-eq/kg NH₃ for SMR and 0.37 to 1.11 kg CO₂-eq/kg NH₃ for the retrofit. Under equal leakage assumptions, the retrofit remains lower than the SMR; however, absolute values depend on leakage and region.

These results suggest that integrated reforming systems with carbon valorization, such as the one modeled in this study, may offer a viable pathway for decarbonizing ammonia production by coupling CO₂ reactivity with the formation of durable carbon products. However, the long-term carbon permanence and scalability of such pathways require further assessment, which is beyond the scope of this work.

### Process integration and infrastructure compatibility

Beyond energy and emissions metrics, the practical feasibility of deploying low-carbon reforming configurations within existing ammonia plants is a critical consideration in evaluating their scalability. The retrofitted configuration modeled in this study exhibits a high degree of compatibility with conventional ammonia plant infrastructure. Following the retrofit, no substantial downstream modifications are required beyond the reforming section, as all subsequent units, including high- and low-temperature shift reactors, CO₂ removal systems, methanation, and the ammonia synthesis loop, continue to operate under pressure-temperature conditions comparable to those in the baseline SMR configuration. This compatibility facilitates integration with minimal capital disruption and preserves established process control schemes.

In addition to maintaining process continuity, the modeled retrofit offers potential thermal integration advantages through its autothermal operation. By eliminating the need for fired reformer furnaces typically required in SMR systems, this approach reduces dependence on external fuel combustion and enhances overall thermal efficiency.

However, the shift away from flue gas combustion also necessitates a reconfiguration of the plant’s heat recovery strategy. In SMR-based systems, significant heat recovery is achieved using process gas preheaters and flue gas-based Waste Heat Recovery Units (WHRUs), which are not applicable in the retrofitted configuration. Instead, heat recovery is maintained via internal boiler and exchanger configurations within the reforming section, with adjustments to boiler duty and exchanger sizing tailored to the altered thermal profile.

In this study, autothermal operation reduces fired/steam duty and onsite steam generation while adding modest compressor loads, yielding a net decrease in total specific energy (see Tables S1–S3). On capital scope, the removal of fired reformers is offset by the addition of dual-reactor hardware, CO₂ compression/recycle, and MWCNT recovery/handling, resulting in a + 50% central CAPEX uplift with a + 35–65% range (Table S5). On operating costs, the mix shifts: fuel/steam components decrease, electricity and oxygen supply increase (purchased O₂ baseline; ASU sensitivity), and feedstock costs rise with higher methane throughput (Table 3; Tables S6a–S6b).

Overall, the reconfiguration lowers total energy demand and rebalances cost structure without requiring major downstream changes to shift/CO₂ removal/methanation/Haber–Bosch. The case study indicates that integrated reforming systems can be implemented with minimal infrastructure changes while maintaining operational continuity and improving energy efficiency, subject to the design constraints and boundary conditions modeled here.

### Techno-economic comparison

To assess the economic viability of the modeled retrofitted configuration using dual-reactor reforming, a comprehensive techno-economic analysis was conducted. This evaluation extends beyond conventional cost metrics by incorporating multiple financial indicators, namely, the LCOA, NPV, IRR, and payback period. Additionally, a sensitivity analysis was conducted to examine the impact of key market drivers, including the prices of MWCNTs, carbon credits, ammonia, and natural gas. Together, these metrics provide a comprehensive view of the comparative performance of the baseline and retrofitted ammonia production scenarios under both baseline and variable conditions. At equal capacity and cost year (2022 USD), the baseline SMR CAPEX used in the analysis is $454.3 million (Table S4), while the retrofitted configuration, with + 50%, adopts a central CAPEX of $681.4 million (Table S5). Consistent with the energy shifts in Sect. "Process integration and infrastructure compatibility", OPEX composition also changes: fuel/steam duty decreases, compressor electricity increases, oxygen appears as a purchased utility in the central case (Table 3), and methane feed is higher; maintenance (3% of FCI) and operating labor (10% of OPEX) resulting in an annual OPEX of $88.65 Million, and $247.57 Million for the SMR base case and retrofitted configuration respectively.

#### Levelized cost of ammonia (LCOA)

The LCOA was calculated to represent the average cost per ton of ammonia produced over the project lifetime, accounting for CAPEX and OPEX. For the SMR-based configuration, the LCOA was estimated at $308.3/ton NH₃, which aligns well with values reported for large-scale U.S.-based ammonia plants using SMR and ATR configurations^54^.

In contrast, the retrofitted configuration exhibited a significantly higher LCOA of $736.3/ton NH₃, driven by a 50% CAPEX uplift and substantially elevated operating costs associated with electricity consumption, oxygen supply, and chemical handling for CO₂ utilization and nanomaterial recovery, as supported by earlier GTL case studies^28,33^.

While LCOA remains a widely cited benchmark, it does not capture the environmental and economic co-benefits of emerging decarbonization technologies. Specifically, co-product revenue streams such as MWCNTs and carbon credits, modeled in the retrofit case, are excluded from LCOA calculations. Thus, LCOA alone may undervalue multi-output decarbonization pathways, especially those involving carbon valorization. To address this limitation, additional metrics, including NPV, IRR, and carbon abatement cost, are used to provide a more comprehensive assessment of long-term value.

To quantify the environmental cost-effectiveness of retrofitting, Table 7 presents the estimated cost of carbon abatement. Under the modeled conditions, the retrofit case achieves a 76% reduction in total emissions at an incremental cost of $4.99 billion over 30 years, resulting in an abatement cost of $224.8 per ton CO₂-eq avoided. This value exceeds the EU Emissions Trading System (EU ETS) price, which averaged approximately $85–$100 per ton in 2025, and the U.S. compliance credit values range between $30–$60 per ton of CO₂^43,54^, indicating that the retrofit sits above current policy prices but within the range considered for deep-decarbonization investments.

**Table 7:** Comparative lifetime costs and emissions for SMR and retrofitted configuration ammonia production Pathways.

Metric	SMR-based process	Retrofitted configuration	Difference
Total 30-Year Cost (Million USD)	3,113.8	8,108.6	+ 4,994.8
Total 30-Year CO₂ Emissions (Million tons CO₂-eq)	29.5	7.3	−22.2
Carbon Abatement Cost (USD/ton CO₂-eq avoided)	-	-	224.8

Although the current analysis does not account for tax incentives, it is notable that recent U.S. policy instruments, such as 45 V, significantly enhance the economic competitiveness of blue and hybrid ammonia pathways^54^.

#### Profitability and economic performance

Although LCOA favors the SMR route, a complete profitability analysis reveals a different outcome under the modeled conditions. A discounted cash flow (DCF) model was developed for both systems over a 30-year project horizon, using an 8% discount rate and inflation assumptions of 2% for ammonia prices and 2.5% for OPEX. Cash-flow inputs use the CAPEX values summarized above (SMR $454.3 M; retrofit central $681.4 M) and the OPEX structure from Table 3 and S9 (steam generated on site from natural gas; electricity and oxygen as priced utilities in the central case; maintenance 3% of FCI; operating labor 10% of OPEX).

The ammonia production rate was fixed at 418,440 tons/year, with baseline ammonia revenue of $188.3 million/year (at $450/ton). For the retrofitted configuration, two additional revenue streams were included:

- Carbon credits: Based on avoided emissions of 1.77 kg CO₂-eq/kg NH₃, monetized at $30/ton CO₂, contributing ~$22.2 million/year.
- MWCNT sales: With a modeled yield of 0.19 kg/kg NH₃ and a conservative 0.25 correction factor applied to account for commercialization constraints, the marketable output (~ 79,500 tons/year) was valued at $5/kg, generating approximately $397.5 million/year in potential revenue.

Under these assumptions, the profitability indicators improve significantly for the retrofit case:

- NPV increases from $0.843.3 billion (SMR) to $3.08 billion (Retrofitted configuration).
- IRR rises from 23% to 52%.
- Payback period is reduced from 14.5 to 4.5 years.

**Fig. 4:**
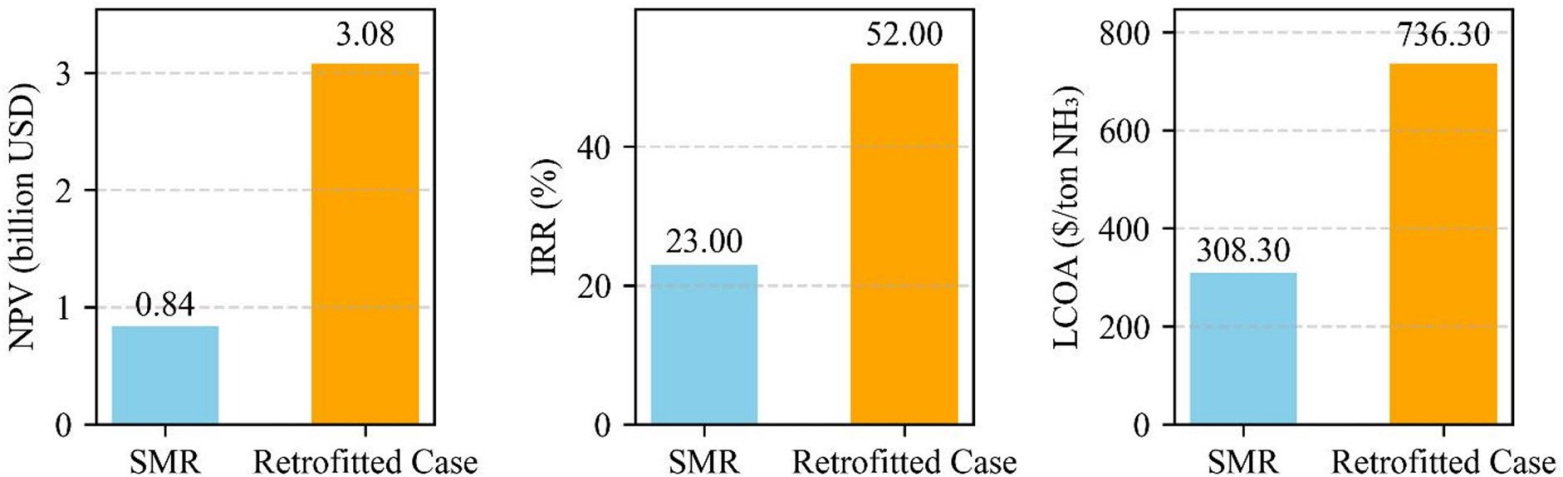
Comparison of Economic Metrics for SMR and Retrofitted Configuration Ammonia Production. *(Ammonia = $450/ton*,* MWCNT = $5/kg*,* Carbon credit = $30/ton CO₂*,* Natural Gas = $4/MMBtu)*.

These results, illustrated in Fig. 4, suggest that integrating a carbon-valorizing reformer may offer long-term economic benefits, particularly when co-products and emission-related incentives are monetized.

Co-product revenue is constrained by exogenous market-absorption caps^55,56^. Under these caps, absolute economic metrics are attenuated relative to the uncapped case because technical output far exceeds near-term demand. The qualitative conclusion remains that co-product revenue materially improves the retrofit’s economics when offtake materializes; however, near-term viability becomes a function of demand development (applications, supply-chain qualification, and offtake contracting).

#### Sensitivity analysis

To better understand the influence of individual market variables on the economic performance of the retrofitted configuration, a sensitivity analysis was conducted across four key factors: MWCNT price, carbon credit value, ammonia price, and natural gas cost. These parameters were selected due to their high volatility and substantial impact on revenue and profitability projections. Sensitivity trends observed in this study reflect those reported in recent techno-economic assessments of blue and hybrid ammonia pathways^57^, particularly regarding the pronounced effect of natural gas price fluctuations on process viability.

##### MWCNT price sensitivity

MWCNT prices vary significantly by purity, grade, and application, with literature citing a wide range from $3/kg to over $1,000/kg^45,58–61^. Sensitivity analysis was conducted across a realistic range of $2 to $80/kg to assess the impact on NPV and IRR.

**Fig. 5:**
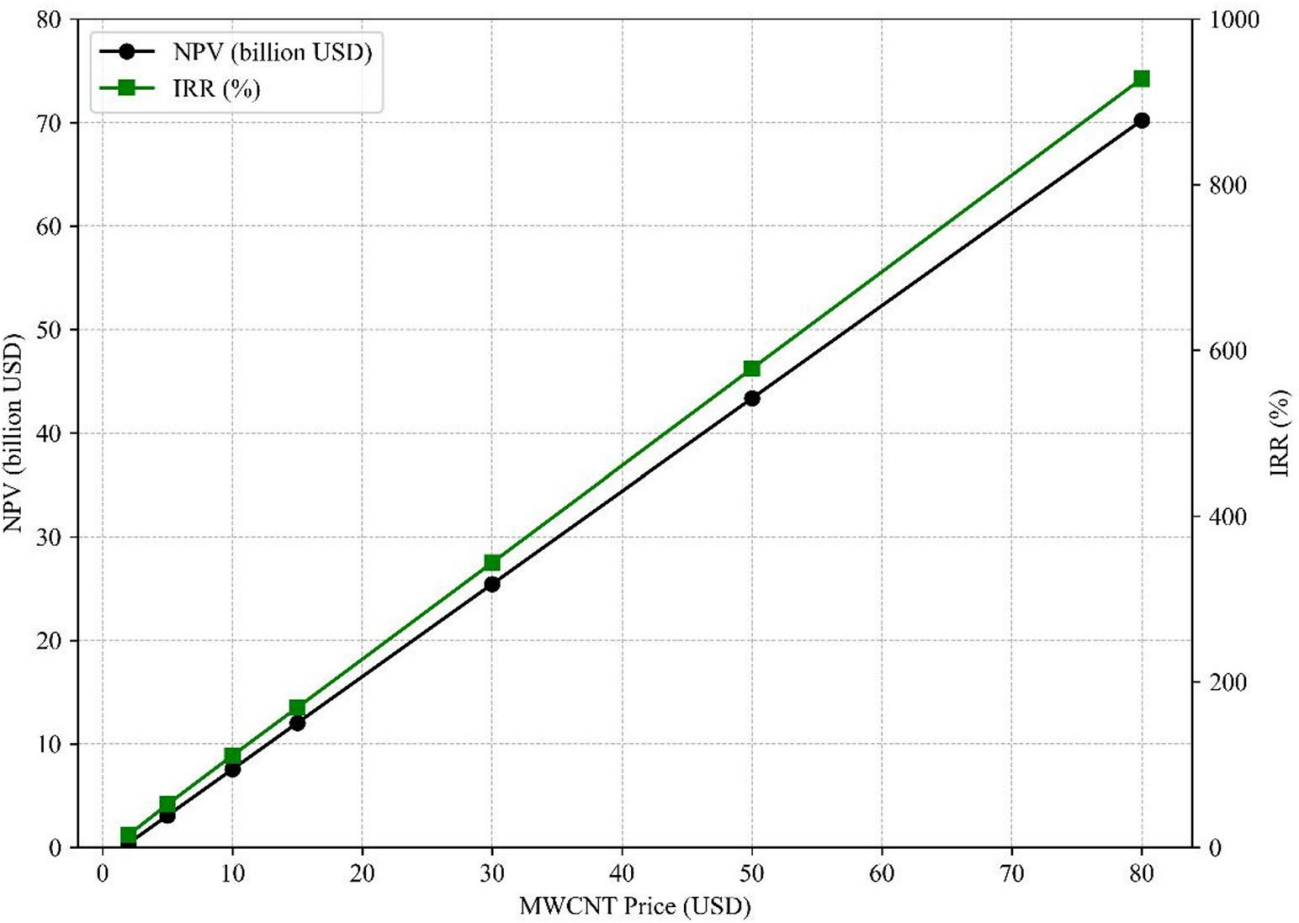
Sensitivity of NPV and IRR to MWCNT Price for the Retrofitted Configuration (Ammonia = $450/ton, Carbon credit = $30/ton CO₂, Natural Gas = $4/MMBtu).

As shown in Fig. 5, both metrics rise steeply with MWCNT price. At $2/kg, the retrofitted configuration still achieves a positive NPV of $0.73 billion and an IRR of 20%. At $50/kg, the NPV exceeds $43 billion, and IRR surpasses 580%; however, such values represent upper-bound theoretical estimates and assume complete market absorption of the co-product at premium prices.

The assumed $5/kg price reflects a conservative estimate for industrial-grade applications such as polymer additives, coatings, and conductive fillers^45,58^. Large-scale deployment would require securing demand in bulk markets such as cement or asphalt, which may offer lower prices but significant volumes.

*Market-absorption sensitivity*: Annual MWCNT sales are capped to external demand scenarios: business-as-usual (BAU) (tens of tons/year with moderate compound annual growth rate (CAGR) of 15% and a high-growth path (hundreds of tons/year by early 2030 s)^55,56^. These caps reduce near-term revenue relative to technical yield. Anticipated uptake spans conductive additives in elastomers and polymers^62^, ESD/antistatic coatings^63^, battery/conductive-polymer systems^64^, and, at lower price points, bulk modifiers for asphalt and cement^65,66^. Realization of high-growth trajectories depends on dispersion/compounding capability, EHS/regulatory acceptance, and application-specific qualification. For consistency with bulk applications, the base case assumes industrial-grade MWCNT pricing; revenue is further constrained by the sales caps (Table S9). In present CNT markets, higher quality/purity (e.g., high-purity MWCNT) commands higher unit prices but is constrained to lower absorptive volumes, whereas industrial-grade MWCNT clears at lower prices in applications capable of absorbing substantially greater tonnage.

##### Carbon credit sensitivity

Carbon credit values varied from $30 to $100 per ton of CO₂. Results show a modest effect: NPV increased from $3.08 to $3.70 billion, and IRR from 52% to 60%. This indicates that while carbon credits improve returns, the economic feasibility of the retrofitted configuration does not depend on high carbon pricing, which is a valuable attribute in uncertain policy environments.

##### Ammonia price sensitivity

**Fig. 6:**
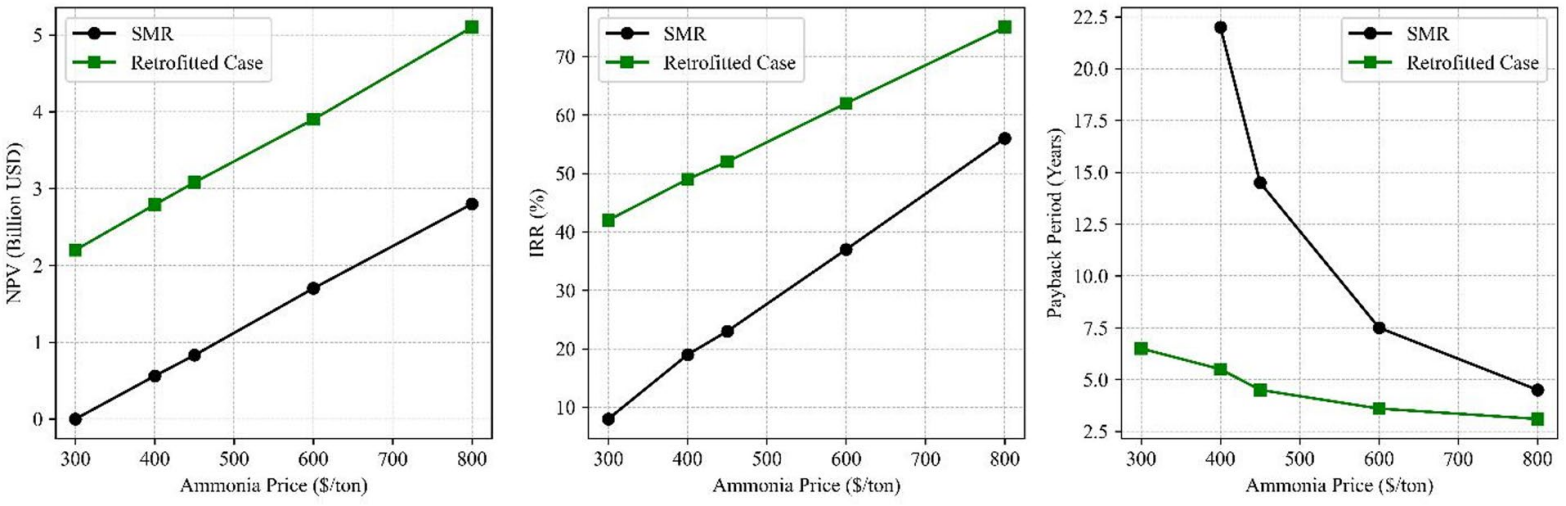
Sensitivity of NPV, IRR, and Payback to Ammonia Price for SMR and the Retrofitted Configuration *(MWCNT = $5/kg*,* Carbon credit = $30/ton CO₂*,* Natural Gas = $4/MMBtu).*

Ammonia prices fluctuate considerably depending on regional energy dynamics and international supply-demand balances, with recent global prices ranging from $300 to over $800 per ton^67,68^. Sensitivity analysis conducted across this range revealed strong profitability trends for both the SMR and retrofitted configurations, as illustrated in Fig. 6.

For the SMR pathway, the NPV increases from $0.02 billion at $300/ton to $2.8 billion at $800/ton, with the IRR rising from 8% to 56%, and the payback period shortening from more than 22 years to 4.5 years. In comparison, the retrofitted configuration demonstrates even greater resilience: its NPV improves from $2.2 billion to $5.1 billion, its IRR increases from 42% to 75%, and its payback period declines from 6.5 years to just 3 years over the exact price interval.

Notably, the retrofitted system remains economically viable even at the low end of the price spectrum, $300/ton, which is well below its calculated LCOA of $736.3/ton NH₃. This continued profitability is made possible by its diversified revenue streams from MWCNT sales and carbon credits, highlighting the limitations of relying solely on LCOA when evaluating low-carbon ammonia technologies.

##### Natural gas price sensitivity

Natural gas prices varied from $1.50 to $13.00/MMBtu, reflecting global conditions, from subsidized gas in the Middle East to high import prices in Europe^38,69–71^.

Table 8 summarizes the impact on economic performance for the retrofitted configuration. The system remains economically viable up to $10/MMBtu, beyond which the project becomes infeasible, with negative NPV and no payback within the 30-year timeframe. This emphasizes the importance of feedstock price stability when deploying reforming technologies with high methane input requirements, particularly in regions aiming for deep decarbonization.

**Table 8:** Impact of natural gas price on the economic viability of the retrofitted configuration *(Ammonia = $450/ton*,* MWCNT = $5/kg*,* carbon credit = $30/ton CO₂).*

Natural gas price ($/MMBtu)	NPV (Billion USD)	IRR (%)	Payback period (Years)
1.5	4.23	65	3.5
3.0	3.74	57	4.1
4.0	3.08	52	4.5
5.0	2.62	47	5.1
7.0	1.69	37	7.2
10.0	0.3	18	> 30
13.0	< 0	-	Not recoverable

##### Oxygen supply option (on-site ASU vs. purchased O₂)

Using an on-site ASU increases CAPEX by + 6.7% and lowers OPEX by 4.4%, yielding LCOA = $710.6/ton (− 3.5% vs. baseline) and NPV = $3.19 billion (+ 3.5%), while IRR decreases from 52% to 22% and payback lengthens from 4.5 y to 5.0 y. This reflects the trade-off between lower unit operating cost and higher capital intensity (Table S6b).

Structural/design sensitivities (CAPEX uplift, maintenance, operating labor, and consumables) were evaluated around the purchased-O₂ baseline; detailed outcomes are in Table S10. In brief, capital intensity dominates rate-based returns, whereas plausible variations in maintenance, labor, and consumables shift NPV/IRR by 3–5% within the tested ranges.

## Conclusions

This study investigates the technical, environmental, and economic performance of retrofitting conventional ammonia plants with an advanced dual-reactor reforming system that integrates CO₂ utilization and carbon valorization. Through steady-state process simulation and techno-economic analysis, the retrofitted configuration achieved a 76% reduction in plant-level (Scopes 1–2) CO₂-equivalent emissions and an 18.2% decrease in total specific energy, and a 31.5% reduction in thermal/steam duties. compared to a traditional SMR-based process, while maintaining the same ammonia production capacity of 1,268 tons per day.

While the LCOA for the retrofitted case is higher than for SMR ($736.3/ton vs. $308.3/ton NH₃), additional revenue streams from co-product recovery, including carbon credits and MWCNTs, significantly improve the economic outlook. The resulting NPV exceeds $3.08 billion, with an IRR of 52% and a payback period of 4.5 years.

Sensitivity analysis confirms that the configuration remains economically viable under conservative pricing scenarios (e.g., $300/ton ammonia and $2/kg MWCNTs), indicating resilience to market variability.

The findings support the feasibility of integrating carbon conversion into existing hydrogen-intensive chemical processes as a scalable approach to decarbonization. The dual-reactor reforming concept may be particularly relevant for regions with access to low-cost methane, concentrated CO₂ sources, and carbon offset markets. By reducing emissions and enhancing energy efficiency, this approach directly contributes to global climate goals and aligns with Sustainable Development Goal 7 (Affordable and Clean Energy).

Future research should incorporate a comprehensive LCA, dynamic simulation, and pilot-scale testing to validate performance under operational conditions. Broader applicability may extend to methanol, synthetic fuels, and clean hydrogen production, advancing resource-efficient and low-carbon industrial pathways.

## Supplementary Information

Below is the link to the electronic supplementary material.

Supplementary Material 1

## Data Availability

All data generated or analyzed during this study are included in this published article and its Supplementary Information file.
