# Supplementary material for: Decarbonizing ammonia synthesis plants through retrofitting novel reformer technology
Source: Sci Rep. 2025 Dec 15;16:661. doi: 10.1038/s41598-025-28598-y (PMC12780061; doi:10.1038/s41598-025-28598-y)
Supplement: Supplementary file 1 — Supplementary Material 1 [file 41598_2025_28598_MOESM1_ESM.docx]

**Decarbonizing Ammonia Synthesis Plants Through Retrofitting Novel Reformer Technology**

Tagwa Musa^1*^, Nada Mahmoud^1*^, Mohamed S. Challiwala^1^, Eiman Mohamed^1^, Hanif Choudhury^1^, Nimir O. Elbashir^1,2$^

^1^Chemical Engineering Program, Texas A&M University at Qatar, 23874 Doha, Qatar

^2^College of Science and Engineering, Hamad bin Khalifa University, Doha, Qatar

*Equal first author contribution

[^$^nelbashir@tamu.edu](mailto:$nelbashir@tamu.edu)


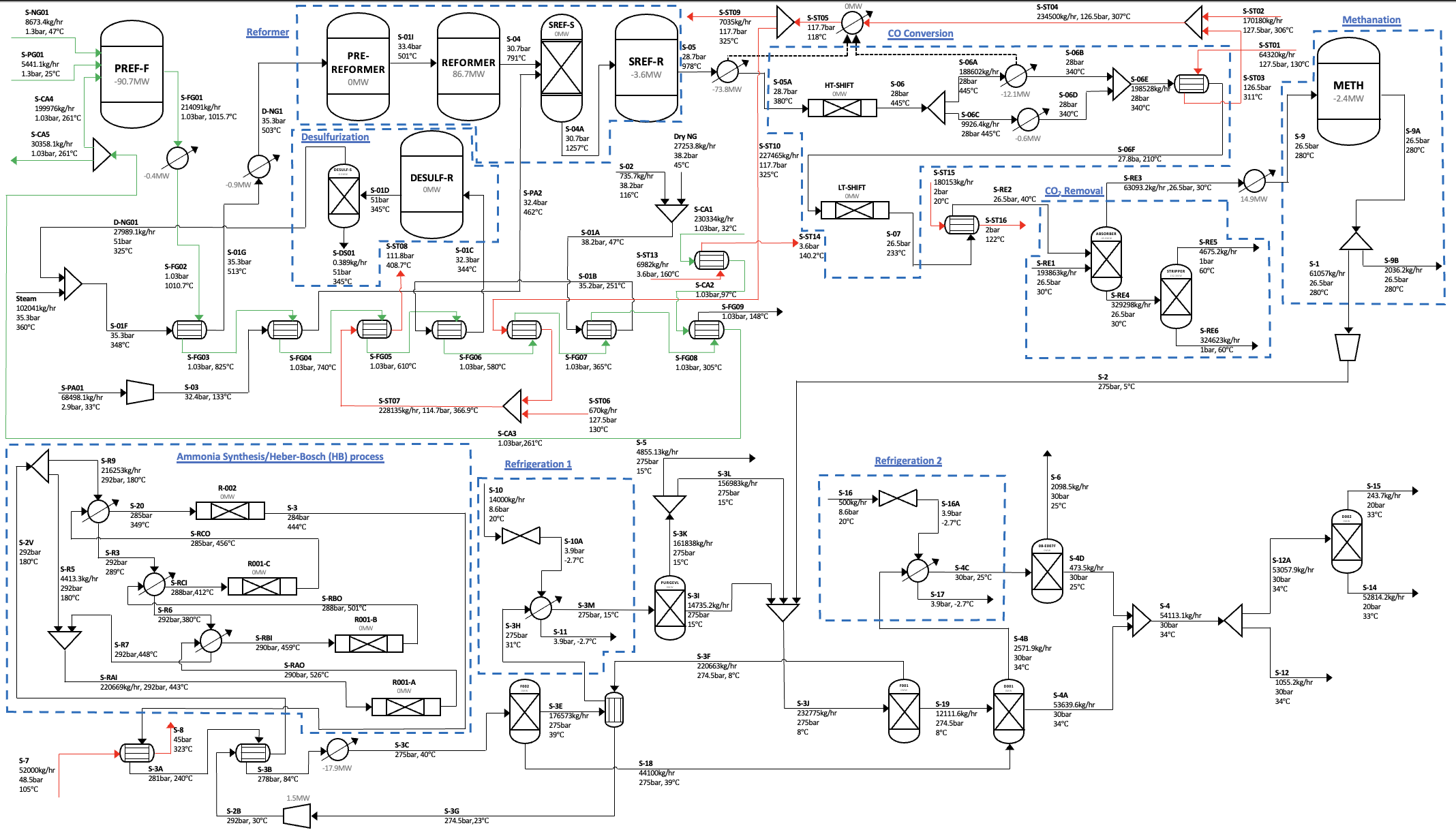


**Fig S1a:** Detailed flowsheets of SMR base case with full stream tags


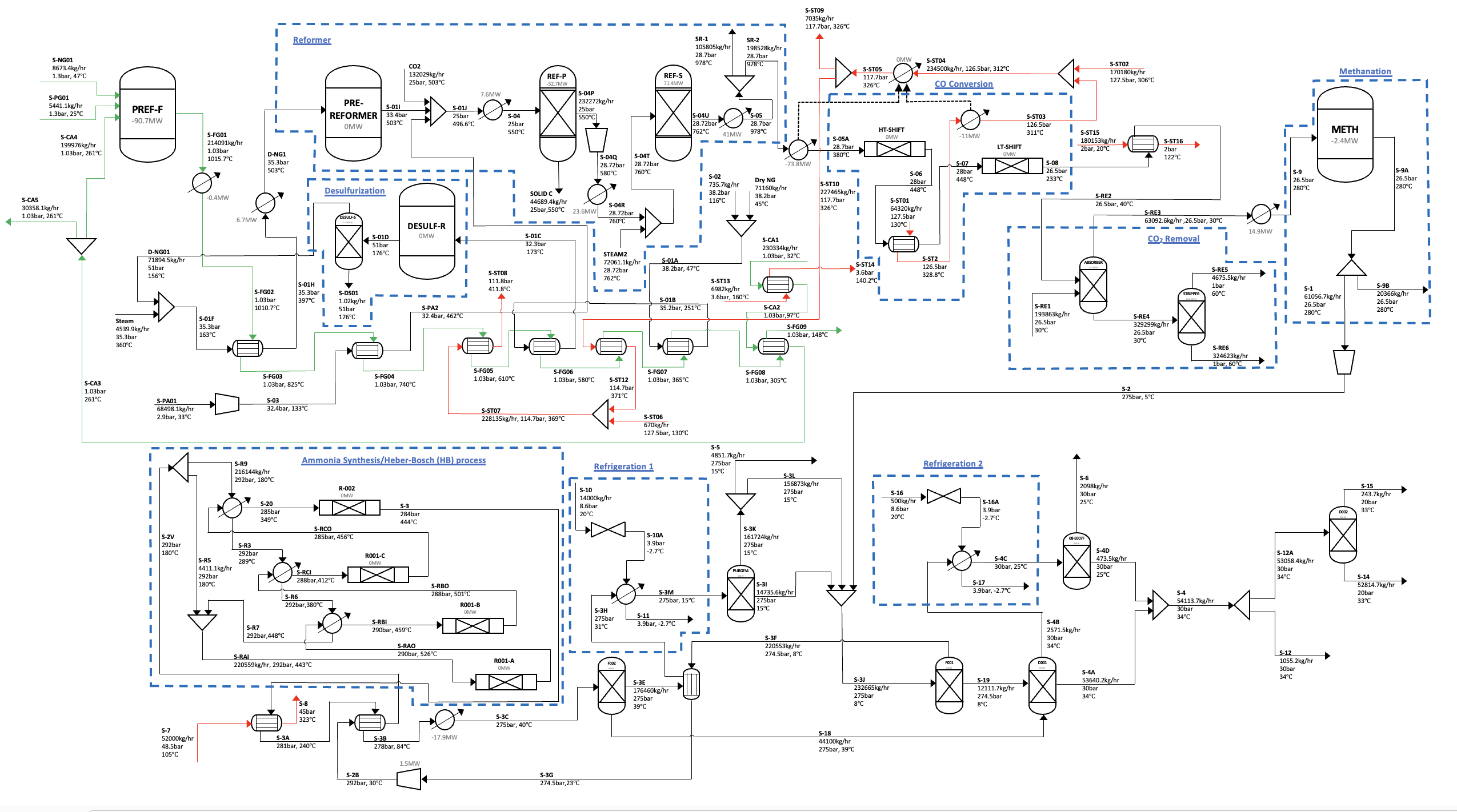


**Fig S1b:** Detailed flowsheets of retrofitted scenario with full stream tags

**Table S1.** SMR case – Stream-by-stream mass & state variables (per kg NH₃).

| **Block / Stream** | **Flow (kg/ kg NH₃)** | **T (°C)** | **P (bar)** | **Key composition (mol %) / Notes** |
| --- | --- | --- | --- | --- |
| Natural gas feed (to desulfurization) | 0.52 | 45 | 38.2 | CH₄ 80.0; C₂H₆ 17.7; C₃+ 1.25; N₂ 0.8; O₂ 0.2; S 1 × 10⁻⁴ |
| Steam to primary reformer | 1.93 | - | - | Saturated HP steam (utility model conditions) |
| Primary reformer outlet | - | 791 | 30.7 | Syngas: H₂ ≈ 58; CO ≈ 8; CH₄ ≈ 0.5; CO₂ ≈ 3; H₂O ≈ 30 (Ni catalyst) |
| Secondary reformer outlet | - | 980 | 29 | H₂ 35.5; H₂O 35.3; N₂ 15.2; CO 8.4; CO₂ 5.1; CH₄ 0.3; Ar 0.2 |
| WGS outlet | - | ≈ 233 | ≈ 26.5 | CO 0.2; CO₂ ≈ 15; H₂ ≈ 65; H₂O ≈ 19; N₂ ≈ 1 |
| CO₂-removal outlet | 1.19 | ≈ 30 | ≈ 26 | CO₂ < 0.3; H₂ ≈ 74; N₂ ≈ 25; CH₄ < 1 |
| Methanation outlet (HB feed) | - | ≈ 280 | ≈ 26.5 | H₂ 74; N₂ 25; CH₄ < 1 (trace CO, CO₂ removed) |
| Haber–Bosch loop | - | - | ≈ 292 | Single-pass N₂ conversion ≈ 24 %; purge ≈ 3 % |
| NH₃ product | 1.00 | - | - | 99.9 wt % NH₃ |

**Table S2.** Retrofitted case – Stream-by-stream mass & state variables (per kg NH₃).

| **Block / Stream** | **Flow (kg/kg NH₃)** | **T (°C)** | **P (bar)** | **Key composition/ratios** |
| --- | --- | --- | --- | --- |
| Dual-reactor feed (R1 basis) | - | R1 ≈ 550; R2 ≈ 760 | 25 (R1); ≈ 28.7 (R2) | CH₄:CO₂:O₂ = 1:0.6:0.1 (mol) |
| Process natural gas to plant | 1.36 | - | - | Increased feed to sustain syngas + carbon formation |
| Process steam | 1.45 | - | - | Reduced vs SMR |
| CO₂ feed (to R1/R2) | ≈ 1.44 | - | - | External CO₂ utilization input |
| R2 (retrofitted reformer) outlet | - | ≈760 | ≈28.7 | H₂ ≈ 60; CO ≈ 7; CO₂ ≈ 6; CH₄ ≈ 0.5; H₂O ≈ 26; solid C ≈ 0.5 wt% |
| Carbon formed (at battery limits)* | 0.76 | - | - | MWCNTs produced in R1/R2 |
| NH₃ product** | 1.00 | - | - | 99.9 wt % NH₃ |

*For TEA, a 25% collection factor is applied to the formed carbon, giving 0.19 kg/kg NH₃ “marketable” MWCNTs; the 0.76 kg/kg NH₃ quantity is the material balance at battery limits.

**Downstream WGS/CO₂-removal/methanation/HB operate at the same T–P targets and specifications as in Table S1.

**Table S3.** Unit-operation energy breakdown (kWh/kg NH₃)

*Electricity (from compressor power)*

| **Machine** | **SMR (kWh/kg NH₃)** | **Retrofitted (kWh/kg NH₃)** |
| --- | --- | --- |
| Air compressor | 0.12 | 0.11 |
| Ammonia compressor A | 0.40 | 0.40 |
| Ammonia compressor B | 0.028 | 0.03 |
| CO₂ compressor | — | 0.44 |
| Retrofitting compressor | — | 0.07 |
| Total electricity | 0.54 | 1.04 |

*Heat / steam-equivalent (reactors, heat exchangers, separators)*

| **Category** | **SMR (kWh/kg NH₃)** | **Retrofitted (kWh/kg NH₃)** |
| --- | --- | --- |
| Reactors (duty total) | 4.15 | 2.58 |
| Heat exchangers (duty total) | 0.37 | 0.52 |
| Subtotal (thermal) | 4.52 | 3.10 |

***Steam costing and conversion basis.***

The on-site steam unit cost is derived from a first-principles heat balance and fuel price:

$$C_{steam}=\frac{\Delta h_{steam\leftarrow feedwater}}{\eta_{boiler}}\times\frac{1}{{HHV}_{NG}}\times P_{NG}$$

Where $\Delta h_{steam\leftarrow FW}$ is the specific enthalpy rise from deaerated feedwater to the delivered HP steam, $\eta_{boiler}$is boiler efficiency, ${HHV}_{NG}$is the higher heating value of natural gas, and $P_{NG}$ is the NG price.

Using $\Delta h=2.9GJ/ton$, $\eta_{boiler}=0.90$^34,46^, ${HHV}_{NG}=1.055 GJ/MMBtu$, $P_{NG}=\$4/MMBtu$^34^, gives $12.2/ton of steam. When $P_{NG}$ varies (Table 3), $C_{steam}$ scales linearly.

***Steam-to-energy conversion (used for validation of Table S3/S7):***

$$E_{steam}\left( \frac{kWh}{Kg NH_{3}} \right)=s\left( \frac{Kg steam}{Kg NH_{3}} \right)\times\frac{\Delta h_{steam\leftarrow FW}}{3.6MJkWh^{-1}}\times\frac{1}{1000}\left( \frac{ton}{Kg} \right)$$

With $\Delta h$ =2.9 GJ/ton ⇒ 0.806 kWh per Kg of steam

SMR: s=1.93 ⇒ $E_{steam}$=1.56 kWh/Kg NH_3_

Retrofit: s=1.45 ⇒$E_{steam}$ =1.17 kWh/Kg NH_3_

**Table S4.** SMR CAPEX back-calculation worksheet (2022 USD). Basis: LCOA from Lee et al.^29^, plant capacity 1,268 ton NH₃/ day.

| **Quantity** | **Symbol/equation** | **Value** | **Unit/note** |
| --- | --- | --- | --- |
| Levelized cost of ammonia (baseline) | LCOA | 229.0 | $ per t NH₃ (Lee et al., 2022) |
| Annual NH₃ production | Q | 418,440 | ton per year (1,268 ton/d × 330 d/y) |
| Discount rate; project life | r; n | 8; 30 | % ; years |
| Capital recovery factor | CRF = r(1+r)^n^ / ((1+r)^n−1^) | 0.088827 | — |
| Implied OPEX (per ton) | c_opex_ | 132.6 | $ per ton NH₃ |
| Annual OPEX | C_opex_ = c_opex_ × Q | 55.49 | M$ per year |
| Back-calculated CAPEX | CAPEX = (LCOA×Q − C_opex_)/CRF | 454.3 | M$ (2022 USD) |

**Table S5**. Retrofit CAPEX rationale — displaced vs. added sections and indicative contributions at 1,268 ton NH₃/day.

| **Scope/Area** | **Item** | **SMR base** | **Retrofit** | **CAPEX effect (Δ vs SMR total)** | **Rationale / Notes** |
| --- | --- | --- | --- | --- | --- |
| **Sections displaced (credit vs. SMR)** | |  |  |  |  |
| Front-end reforming | Fired primary reformer (tubular furnace) | Present | Removed | −10% to −20% | Large fired box and radiant coils removed; some heat integration retained by new exchangers. |
| Front-end reforming | Secondary reformer (air addition) | Present | Removed | −3% to −6% | Eliminated with O₂-assisted advanced reforming; N₂ supplied via ASU interface. |
| **Sections added (debits vs. SMR)** | |  |  |  |  |
| Advanced reforming (ISBL) | Reactor 1 (carbon-forming) + solids-compatible heat train | Absent | Added | +15% to +25% | Carbon-forming reactor vessel/internals; moderate-T exchangers, quench/knockout. |
| Advanced reforming (ISBL) | Reactor 2 (high-T reformer) + preheat train | Absent | Added | +10% to +18% | High-T syngas conditioning; increased preheat duty. |
| CO₂ handling (ISBL/OSBL) | CO₂ compression, recycling, and polishing | Absent | Added | +4% to +8% | Recycle/bleed compression; tie-ins to AGR; controls/piping. |
| Carbon product handling (ISBL) | MWCNT separation/collection, interim storage | Absent | Added | +3% to +7% | Solids disengagement/collection; contained handling. |
| Controls & interconnects (OSBL) | Piping, E&I, control expansion | Baseline | Expanded | +2% to +5% | Additional loops, analyzers, and safeguarding for dual-reactor and CO₂/O₂ service. |

*Note: Percentages indicate the CAPEX effect relative to the total SMR base-plant CAPEX (ISBL+OSBL). The central estimate (+50%) is used in the main case, while a range of +35% to +65% is used in sensitivity analysis.*

**Table S6a.** ASU sensitivity (cryogenic) at 1,268 t NH₃/day.

Specific power = 220 kWh/ton O₂ (range 200–400 kWh per ton O*^52^*. Indirect emissions factor 0.36 kg CO₂/ kWh. Electricity price $0.07/kWh.

| **Quantity** | **Formula** | **Central** | **Range** | **Units** |
| --- | --- | --- | --- | --- |
| NH₃ production rate | - | 1,268 | - | ton NH₃/day |
| O₂ demand (plant) | - | 340.7 | - | Ton O₂/day |
| O₂ per NH₃ | 340.7/1,268 | 0.269 | - | ton O₂/ton NH₃ |
| ASU specific power | - | 220 | 200–400 | kWh/ton O₂ |
| Extra electricity (per ton NH₃) | ASU×0.269 | 59.18 | 53.74–107.48 | kWh/ton NH₃ |
| Extra electricity (plant, daily) | ASU×1268 | 75,040 | 68,140–136,280 | kWh/day |
| Share of plant electricity | ΔE_daily_/1,322,097.36 | 5.68 % | 5.15–10.31 % | - |
| Scope-2 CO₂ (per ton NH₃) | 0.36×ΔE_per-t_ | 21.30 | 19.35–38.69 | kg CO₂/ton NH₃ |
| Scope-2 CO₂ (plant, daily) | 0.36×ΔE_daily_​ | 27.01 | 24.53–49.06 | ton CO₂/day |
| Power OPEX (per ton NH₃) | ΔE_per-t_×$0.07 | $4.14 | $3.77-$7.53 | $/ton NH₃ |
| Power OPEX (annual) | 330×ΔE_daily_×$0.07​ | $1.7 M | $1.57-$3.15 M | $/year |

**Table S6b**. Oxygen supply option — economic comparison (purchased O₂ baseline vs. on-site cryogenic ASU).

Discount rate 8% (real), project life 30 years. Prices and OPEX structure as in Table 3. Purchased O₂ is the baseline.

| **Metric** | **Purchased O₂ (baseline)** | **On-site ASU** | **Δ vs. baseline** |
| --- | --- | --- | --- |
| CAPEX (Million) | $681.45 | $726.83 | +6.7% |
| Annual OPEX (Million) | $247.6 | $236.8 | −4.4% |
| LCOA ($/ton NH_3_) | $736.32 | $710.56 | −3.5% |
| NPV (Billion) | $3.08 | $3.188 | +3.5% |
| IRR (%) | 52 | 22 | −30 percentage points |
| Payback (Years) | 4.5 | 5.0 | +0.5 y |

*Notes. (i) On-site ASU improves unit cost (lower OPEX) and slightly increases NPV, but reduces IRR and lengthens payback due to higher capital intensity. (ii) Incremental ASU power/emissions accounting is detailed in Table S6a (cryogenic specific power 220 kWh/ton O₂; 200–400 kWh/ton O₂ range^52^ (iii) Scope-2 factors and electricity price follow Table 3 and Section 2.6.2.*

**Table S7.** CO₂-equivalent emissions by process step, species, and scope (kg CO₂-eq/kg NH₃)

| Process step/source | Species | Scope | SMR (conv.) | Retrofitted (dual-reactor) |
| --- | --- | --- | --- | --- |
| Net process CO₂ at battery limits (process + combustion combined)* | CO₂ | Direct (Scope 1) | **0.52** | **-0.92** |
| Air compressor | CO₂ from electricity | Indirect (Scope 2) | 0.043 | 0.038 |
| Ammonia compressor A | CO₂ from electricity | Indirect (Scope 2) | 0.143 | 0.143 |
| Ammonia compressor B | CO₂ from electricity | Indirect (Scope 2) | 0.010 | 0.010 |
| CO₂ compressor | CO₂ from electricity | Indirect (Scope 2) | - | 0.158 |
| Retrofit compressor | CO₂ from electricity | Indirect (Scope 2) | - | 0.026 |
| Reactor duties (thermal utilities) | CO₂ from utilities | Indirect (Scope 2) | 1.493 | 0.927 |
| Heat-exchanger duties (thermal utilities) | CO₂ from utilities | Indirect (Scope 2) | 0.133 | 0.187 |
| Subtotal Indirect (electric + thermal) | CO₂ | Indirect (Scope 2) | **1.83** | **1.50** |
| Total (Direct + Indirect) | CO₂-eq | - | **2.35** | **0.58** |
| ASU (Case B sensitivity) | CO₂ from electricity | Indirect (Scope 2) | - | 0.021 |

*Notes.(i) Direct (Scope 1) reflects battery-limit net process CO₂; carbon retained in MWCNTs is counted at battery limits only. (ii) Scope-2 emissions are computed by converting electricity and thermal (steam-equivalent) duties to kWh and applying 0.36 kg CO₂ per kWh; process-step allocations sum to the subtotals shown. (iii) Steam-related indirect emissions use a steam conversion of Δh ≈ 2.9 GJ per t HP steam (≈ 0.806 kWh per kg steam) with the same 0.36 factor, implying ≈ 290 kg CO₂ per t HP steam. (iv) ASU (Case B sensitivity) applies to the retrofitted case only and is based on 200–400 kWh/ton O₂ evaluated with 0.36 kg CO₂/ kWh.*

**Table S8.** Upstream fugitive methane sensitivity (GWP₁₀₀, AR6 fossil CH₄ = 27.2).

| Case | NG feed (kg per kg NH₃) | Leakage (%) | CH₄ leaked (kg per kg NH₃) | Added CO₂-eq (kg per kg NH₃) |
| --- | --- | --- | --- | --- |
| SMR | 0.52 | 1 | 0.0052 | 0.14 |
|  |  | 3 | 0.0156 | 0.42 |
| Retrofit | 1.36 | 1 | 0.0136 | 0.37 |
|  |  | 3 | 0.0408 | 1.11 |

*Method: leakage applied to plant-gate natural-gas mass; treated as CH₄; conversion via GWP₁₀₀ (AR6); leakage range 1–3 % consistent with Sherwin et al.^36^ and Burns & Grubert^37^.*

**CNT demand scenarios & annual sales cap (BAU and High-growth).**

Annual MWCNT sales are capped by exogenous market-absorption scenarios; two cases are reported: BAU (60 ton/y in 2026 with 15% CAGR) and High-growth (100 ton/y in 2026 rising to 800 ton/y by 2035)^55,56^. Technical output after recovery is 79,503.6 ton/y in all years; excess production is unsold (no revenue).

**Table S9a:** CNT demand scenario-Business-as-Usual (BAU); cap starts 60 ton/y in 2026, 15% CAGR^55,56^

| **Year** | **Scenario demand (ton/y)** | **Technical output × recovery (ton/y)** | **Absorbed sales (ton/y)** | **Unsold (ton/y)** | **Price band ($/kg)** | **Revenue ($/y)** |
| --- | --- | --- | --- | --- | --- | --- |
| 2026 | 60 | 79,503.60 | 60 | 79,443.60 | 5 | 300,000 |
| 2027 | 69 | 79,503.60 | 69 | 79,434.60 | 5 | 345,000 |
| 2028 | 79.35 | 79,503.60 | 79.35 | 79,424.25 | 5 | 396,750 |
| 2029 | 91.25 | 79,503.60 | 91.25 | 79,412.35 | 5 | 456,262.5 |
| 2030 | 104.94 | 79,503.60 | 104.94 | 79,398.66 | 5 | 524,701.88 |
| 2031 | 120.68 | 79,503.60 | 120.68 | 79,382.92 | 5 | 603,407.16 |
| 2032 | 138.78 | 79,503.60 | 138.78 | 79,364.82 | 5 | 693,918.23 |
| 2033 | 159.60 | 79,503.60 | 159.60 | 79,344.00 | 5 | 798,005.96 |
| 2034 | 183.54 | 79,503.60 | 183.54 | 79,320.06 | 5 | 917,706.86 |
| 2035 | 211.073 | 79,503.60 | 211.073 | 79,292.53 | 5 | 1,055,362.89 |

*Notes: demand(t) = 60 × 1.15^(year−2026)^*

**Table S9b:** CNT demand scenario -High-growth; cap starts 100 ton/y in 2026, 26% CAGR^55,56^

| **Year** | **Scenario demand (ton/y)** | **Technical output × recovery (ton/y)** | **Absorbed sales (ton/y)** | **Unsold (ton/y)** | **Price band ($/kg)** | **Revenue ($/y)** |
| --- | --- | --- | --- | --- | --- | --- |
| 2026 | 100 | 79,503.60 | 100 | 79,403.60 | 5 | 500000 |
| 2027 | 126 | 79,503.60 | 126 | 79,377.60 | 5 | 630,000 |
| 2028 | 158.76 | 79,503.60 | 158.76 | 79,344.84 | 5 | 793,800 |
| 2029 | 200.04 | 79,503.60 | 200.04 | 79,303.56 | 5 | 1,000,188 |
| 2030 | 252.05 | 79,503.60 | 252.05 | 79,251.55 | 5 | 1,260,236.88 |
| 2031 | 317.58 | 79,503.60 | 317.58 | 79,186.02 | 5 | 1,587,898.47 |
| 2032 | 400.15 | 79,503.60 | 400.15 | 79,103.45 | 5 | 2,000,752.07 |
| 2033 | 504.19 | 79,503.60 | 504.19 | 78,999.41 | 5 | 2,520,947.61 |
| 2034 | 635.28 | 79,503.60 | 635.28 | 78,868.32 | 5 | 3,176,393.99 |
| 2035 | 800.45 | 79,503.60 | 800.45 | 78,703.15 | 5 | 4,002,256.42 |

*Note: (i) The paper’s headline NPV/IRR are reported for the central (uncapped) pricing baseline; demand-capped sales in Table S9 are provided to bound near-term offtake without over-interpreting project finance under transient market constraints. (ii) Price band refers to product grade: base case uses industrial-grade MWCNT at $5/kg for bulk applications; higher-purity MWCNT pricing is higher but applies to lower-volume markets and is not used in the base revenue case.*

**Table S10.** Structural/design parameter sensitivity (one-factor-at-a-time).

| **Parameter (basis)** | **Case** | **NPV (B$)** | **IRR (%)** |
| --- | --- | --- | --- |
| CAPEX uplift vs SMR | +35% | 3.18 | 59 |
|  | +50% (baseline) | 3.08 | 52 |
|  | +65% | 2.98 | 47 |
| Maintenance (% of FCI) | 1.5% | 3.25 | 54 |
|  | 3.0% (baseline) | 3.08 | 52 |
|  | 4.5% | 2.91 | 50 |
| Operating labor (% of OPEX) | 5% | 3.24 | 54 |
|  | 10% (baseline) | 3.08 | 52 |
|  | 15% | 2.92 | 50 |
| Chemicals/consumables (% of OPEX) | 0% | 3.18 | 53 |
|  | 3% (baseline) | 3.08 | 52 |
|  | 6% | 2.98 | 51 |

*Notes. Purchased O₂ baseline: CAPEX $681.5 M; OPEX $247.6 M/y; NPV $3.08 B; IRR 52%; payback 4.5 y. Sensitivities vary one parameter at a time, with all other assumptions following Table 3 and Section 2.7.*

**References**

References are identical to the main text.
